# Low-Grade Chronic Inflammation and Atherosclerotic Cardiovascular Risk: The Beneficial Role of Specific Nutritional Approaches

**DOI:** 10.3390/nu18172834

**Published:** 2026-08-28

**Authors:** Claudia Giglione, Reham Hattab, Hygerta Berisha, Paolo Magni

**Affiliations:** 1 Department of Pharmacological and Biomolecular Sciences “Rodolfo Paoletti”, Universita’ degli Studi di Milano, via Balzaretti 9, 20133 Milano, Italy; claudia.giglione@unimi.it (C.G.); reham.hattab@unimi.it (R.H.); hygerta.berisha@unimi.it (H.B.); 2 Istituto di Ricovero e Cura a Carattere Scientifico MultiMedica, via Milanese 300, 20099 Sesto San Giovanni, Italy

**Keywords:** low-grade chronic inflammation, atherosclerosis, nutrition, cardiovascular disease, high-sensitivity C-reactive protein, nutraceutical product, food supplement

## Abstract

Healthy nutrition is pivotal for the prevention of atherosclerotic cardiovascular disease (ASCVD). In addition to traditional risk factors, other components, like low-grade chronic inflammation, play a pathogenetic role in ASCVD. Research efforts aim to identify low-grade chronic inflammation in patients with established ASCVD and in subjects with low ASCVD risk, and to validate effective nutritional strategies. Here, current nutritional approaches to counteract low-grade chronic inflammation related to ASCVD and possibly mitigated ASCVD risk are discussed, along with related molecular pathways and clinical biomarkers. Low-grade chronic inflammation results from a pro-inflammatory response promoted by large multiprotein complexes (inflammasomes). Within the ASCVD context, the nucleotide-binding oligomerization domain (NOD)-like receptor family pyrin domain-containing protein 3 (NLRP3) inflammasome is the most characterized protein complex, whose activation leads to increased release of downstream effectors (interleukin (IL)-1β, IL-18, IL-6). In the current clinical practice, high-sensitivity C-reactive protein (hsCRP) is used as a circulating biomarker for systemic inflammation, although it is not univocally associated with ASCVD. Some nutritional strategies have been shown effective to mitigate low-grade chronic inflammation and to reduce hsCRP, reducing in some cases ASCVD risk. The Mediterranean Diet and related trials showed that their beneficial effects on cardiovascular disorders could also be related to the anti-inflammatory properties of specific active compounds. Contradictory results have also been obtained, due to lack of standardization and to the specific nature of nutritional studies, which often rely on adherence and compliance of participants. Given the relevance of low-grade chronic inflammation for ASCVD risk, future work will need to increase awareness, identify and validate novel biomarkers, and implement novel effective nutrition strategies.

## 1. Introduction

Healthy nutrition is notoriously well regarded as the pivotal component for promoting human health and, very relevant to our times, for the prevention and mitigation of chronic non-communicable diseases (NCDs). Indeed, NCD prevention and management represent a major challenge worldwide, especially with reference to cancer, neurodegeneration, and cardiovascular diseases (CVDs) [1]. The latter are the most relevant ones from the epidemiological viewpoint, with atherosclerotic CVD (ASCVD) playing a major role [1]. In the context of cardiovascular health, the Life’s Essential 8 criteria do include healthy diet, along with participation in physical activity, avoidance of nicotine, healthy sleep, healthy weight, and healthy levels of blood lipids and glucose, and of arterial pressure [2]. Such considerations underline the high level of complexity of the pathophysiological mechanisms behind ASCVD development, with a peculiar individual interplay between endogenous factors, including sex, and exposomal components to produce or to prevent onset and progression of ASCVD [3,4,5]. In the context of ASCVD risk and development (and any other chronic NCD), low-grade inflammation has been found to show some sex-related peculiarities, although the full picture has not been clarified yet and scattered data are available. Compared to men, women, especially after the menopause, appear to have a less favorable low-grade inflammation profile, with a positive association with development of arterial hypertension and, ultimately, ASCVD [6]. Scarce and controversial data are available in response to anti-inflammatory dietary interventions. For example, a Mediterranean-DASH Intervention for Neurodegenerative Delay Diet Score (MIND-S) showed that higher scores were significantly associated with lower hsCRP concentrations in older community-dwelling Chinese males, but not in females [7]. Moreover, in a Japanese population, hsCRP levels in men were unexpectedly inversely associated with the healthy, bread, and dessert patterns and positively associated with the seafood pattern, while in women, hsCRP levels were inversely associated with the healthy pattern and positively associated with the Western diet pattern [8]. These observations should therefore promote more in-depth research about the sex and age relationship with low-grade inflammation, response to dietary interventions and ASCVD risk. As a consequence, while the available risk algorithms, like the SCORE2 [9], are currently useful in clinical practice, a more accurate prevention and management of ASCVD will require a personalized approach which takes into consideration a multitude of factors, including multiomics parameters, such as, relevant to the topic of this paper, genetic variability related to anti- or pro-inflammatory response to diet, to be integrated with artificial intelligence/machine learning approaches [10,11,12,13]. As these novel approaches may have an economic impact when applied to a population, their actual cost/effectiveness will need to be carefully assessed, and the specific target subpopulation should be properly defined. For example, subjects at high-risk for ASCVD may benefit from a deeper phenotyping according to the above considerations. In this context, the outcomes of lifestyle and pharmacological strategies to mitigate ASCVD risk and progression are currently based on the assessment of traditional risk factors, including LDL cholesterol levels, presence of arterial hypertension, obesity, type 2 diabetes mellitus, as well as the combination of them as diagnostic criteria for the metabolic syndrome, which is recognized as a high-risk condition for ASCVD [14,15]. However, the observation that a residual cardiovascular risk remains after correcting these traditional factors suggested the presence of additional lipid and non-lipid factors able to play some pathogenetic role. The identification of elevated lipoprotein(a) (Lp(a)) is one example of an additional (residual) lipid risk factor [16,17]. Interestingly, low-grade chronic inflammation has also emerged as another pivotal component of the pathogenesis of NCDs and, relevant to this paper, of ASCVD [18]. Higher LDL-C levels, the main ASCVD risk biomarker currently evaluated, were associated with the highest risk for major adverse cardiovascular events (MACE), followed by Lp(a) and hsCRP. One higher standard deviation in LDL-C, Lp(a), and hsCRP was associated with a 13%, 8%, and 6% greater risk for MACEs in non-users and 11%, 7%, and 6% in cholesterol-lowering medication users, respectively. Upon combination, LDL-C, Lp(a), and hsCRP demonstrated a synergistic effect. Compared with individuals with all three biomarkers at or below the 75th percentile, those with all three biomarkers above the 75th percentile had a 77% higher risk for incident MACEs among non-users and a 58% higher risk among those on cholesterol-lowering medications [19]. Recent important research efforts have been implemented to improve the identification of the extent of low-grade chronic inflammation in patients with established ASCVD, as well as in subjects apparently with low ASCVD risk, and to validate nutritional, nutraceutical and pharmacological strategies to mitigate this pathogenetic component of ASCVD risk.

In this narrative review paper, we discuss the scientific evidence regarding the current nutritional and nutraceutical approaches to counteract low-grade chronic inflammation in the context of ASCVD risk and prevention, after providing a short overview of the related molecular pathways and the available clinical biomarkers. As scientific research and clinical awareness about this important pathogenic factor is currently growing, we believe it is useful to highlight the main features related to the role of nutrition in mitigating low-grade inflammation to possibly reduce ASCVD risk.

A search was conducted in the PubMed database using key words such as “nutrition”, “nutraceutical”, “atherosclerotic cardiovascular disease”, “low-grade chronic inflammation”, “high-sensitivity C-reactive protein”, “lipid biomarkers”, “cardiometabolic risk”, “lifestyle”, “bioactive compounds”, “residual cardiovascular risk”, and “lipoprotein (a)”, initially identifying approximately 7000 records according to “best match” selection. To refine the selection, filters were applied for the publication period (2014–2026), yielding 4935 records, English language and full text availability (3512 records), and specific article types (peer-reviewed; limited to adult humans; 1733 records). After further screening according to the specific relevance to the review focus (nutrition + low-grade chronic inflammation + ASCVD), a total of 57 articles were selected for inclusion in this review. We acknowledge some limitations in this selection methodology. Specifically, while nutrition and related guidelines are specifically related to sociocultural, ethnical and geographical issues [20], here we mainly refer to the consolidated evidence developed around structured scientific studies emerged from the Mediterranean area and Western countries.

## 2. Low-Grade Chronic Inflammation: Pathogenic Role in Atherosclerotic Cardiovascular Disease and Diagnostic Approach

In the last 20 years, the role of low-grade silent chronic inflammation has gained high attention in relationship to its relevant pathogenic role in ASCVD onset and progression [18]. Low-grade chronic inflammation is the result of a pro-inflammatory response promoted by large multiprotein complexes defined as “inflammasomes”, which can modulate immuno-inflammatory activity [21]. Several types of inflammasomes have been identified, primarily including scaffold proteins from the nucleotide-binding domain and leucine-rich repeat-containing (NLR) family. These proteins work as pattern recognition receptors (PRRs) able to detect cellular stress signals such as oxidative stress, endoplasmic reticulum stress and mitochondrial dysfunction, as well as cholesterol crystals, bacteria also related to oral dysbiosis and gut microbiotas [22,23], and other danger signals of endogenous and exogenous origin [24]. Within the ASCVD context, the nucleotide-binding oligomerization domain (NOD)-like receptor family pyrin domain-containing protein 3 (NLRP3) inflammasome is the most characterized protein complex [25]. Notably, interleukin (IL)-1β and IL-18, along with IL-6, are some of the main downstream effectors of NLRP3 inflammasome activation [26]. These ILs participate in several and different physiological and pathological pathways, sometimes with bidirectional effects. For example, IL-6 has been described as a cytokine with both beneficial and detrimental effects on the mammalian central nervous system and in other organs [27]. Interestingly, at the clinical level, an additional level of complexity is related to the presence of NLRP3 inflammasome gene variants, which may contribute to increase or reduce the activation of this pathway in response to different pathogenic triggers [24]. Moreover, these specific gene variants are associated with a wider genetic variability that affects the overall inflammatory responses to dietary interventions [28].

Given this complex scenario, an accurate clinical assessment of low-grade chronic inflammation by validated biomarkers remains difficult. Indeed, when elevated, circulating levels of several experimental inflammatory biomarkers, such as serum amyloid A, IL-1b, IL-6, trimethylamine N-oxide (TMAO), fibrinogen, white blood cell count, neutrophil-to-lymphocyte ratio, and eicosapentaenoic acid/arachidonic acid ratio, have been shown to either predict ASCVD risk or be associated with ASCVD [18]. The rationale for assessing plasma IL-1b and/or IL-6 levels in this context is based upon the knowledge that they are the downstream effectors of NLRP3 inflammasome activation, as mentioned above. However, since their pathogenic action may mainly take place within the atherosclerotic plaque rather than systemically, in several cases circulating level changes may not capture such local impact. TMAO is another interesting biomarker, whose circulating levels are the result of a complex process, and, when elevated, are associated with ASCVD risk/development. Briefly, specific dietary components (choline, L-carnitine, phosphatidylcholine, and betaine) are metabolized by some gut bacteria into trimethylamine (TMA), which is then oxidized in the liver into TMAO. Interestingly, TMAO displays pro-inflammatory effects also by activating the NLRP3 inflammasome [26]. Taken together, circulating levels of these molecules, including TMAO, display a large inter-individual variability in the presence of the same ASCVD, possibly due to a subject-specific quantitative and qualitative contribution of low-grade inflammation to ASCVD development. This is clearly making it difficult to even validate reference ranges to be applied in clinical practice [18]. In large clinical trials, these biomarkers have not been proven superior to high-sensitivity C-reactive protein (hsCRP) and many of them are not widely available in standardized commercial formats that may be used in the clinical biochemistry setting rather than in research laboratories [18]. In the future, a composite low-grade inflammation score, including circulating, genomic and imaging biomarkers, could theoretically be useful for the implementation of precision medicine approaches also in this field.

While they still await their validation for diagnostic use [18], some of these molecules (e.g., IL-1b, IL-6) have also represented or represent interesting (and so far experimental) potential pharmacological targets [18].

In the current clinical practice, hsCRP has been proposed and validated, and is used as a circulating biomarker for systemic inflammation, although it is not univocally associated with ASCVD itself. Several important studies took advantage of hsCRP measurement for ASCVD risk stratification and management, reinforcing the evidence of the contribution of low-grade inflammation to disease progression [29].

**Figure 1:**
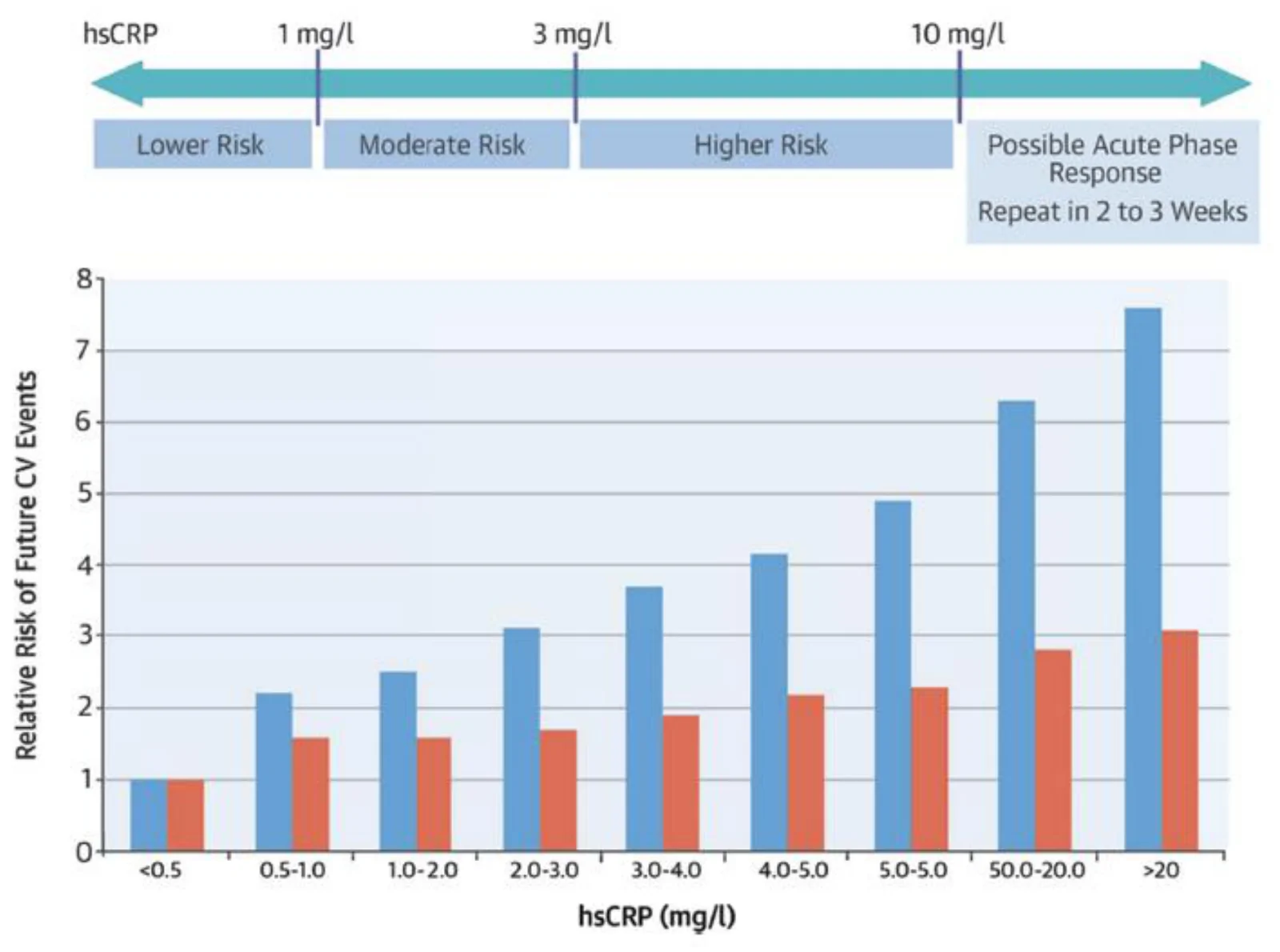
High-sensitivity C-reactive protein (hsCRP) and cardiovascular disease (CVD) risk. The upper panel shows the proposed hsCRP value intervals and the associated CVD risk entity. The lower panel reports the positive association between hsCRP levels and relative risk of future CVD. Blue bars represent crude relative CVD risks; red bars represent relative risks adjusted for traditional Framingham Risk Score Factors. Modified from [31].

Based on these observations, in clinical practice, silent vascular inflammation is commonly evaluated through blood measurement of hsCRP, which adds prognostic information about cardiovascular risk comparable with that of blood pressure and cholesterol. In general, levels of hsCRP < 1, 1 to 3, and >3 mg/L are associated with lower, average, and higher relative cardiovascular risk, respectively, when considered along with integration with other traditional risk factors (Figure 1). As transient infectious processes or other acute-phase responses are associated with elevated levels of hsCRP levels (>10 mg/L), its measurement, when assessed for ACVD risk stratification, should be repeated after such acute events are resolved. Assessment of hsCRP levels may thus help to better stratify ASCVD risk in different subject categories, allowing, for example, to identify patients in secondary CVD prevention with higher risk of additional CVD events, thus prompting the implementation of additional therapeutic strategies [18], and also to identify subjects without standard modifiable risk factors (“SMuRF-less”), but at higher ASCVD risk. To this aim, a recent study conducted in 21,085 SMuRF-less individuals observed that the multivariable adjusted HRs (95% CI) for MACE were 1.35 (1.08–1.68) and 1.36 (1.14–1.62) for the highest vs. lowest clinical threshold and tertile of hsCRP, respectively, suggesting that elevated hsCRP levels are associated with increased CVD risk in (apparently) low-risk women and men [30].

## 3. Nutritional Strategies to Reduce Atherosclerotic Cardiovascular Disease Risk by Mitigating Low-Grade Chronic Inflammation

**Figure 2:**
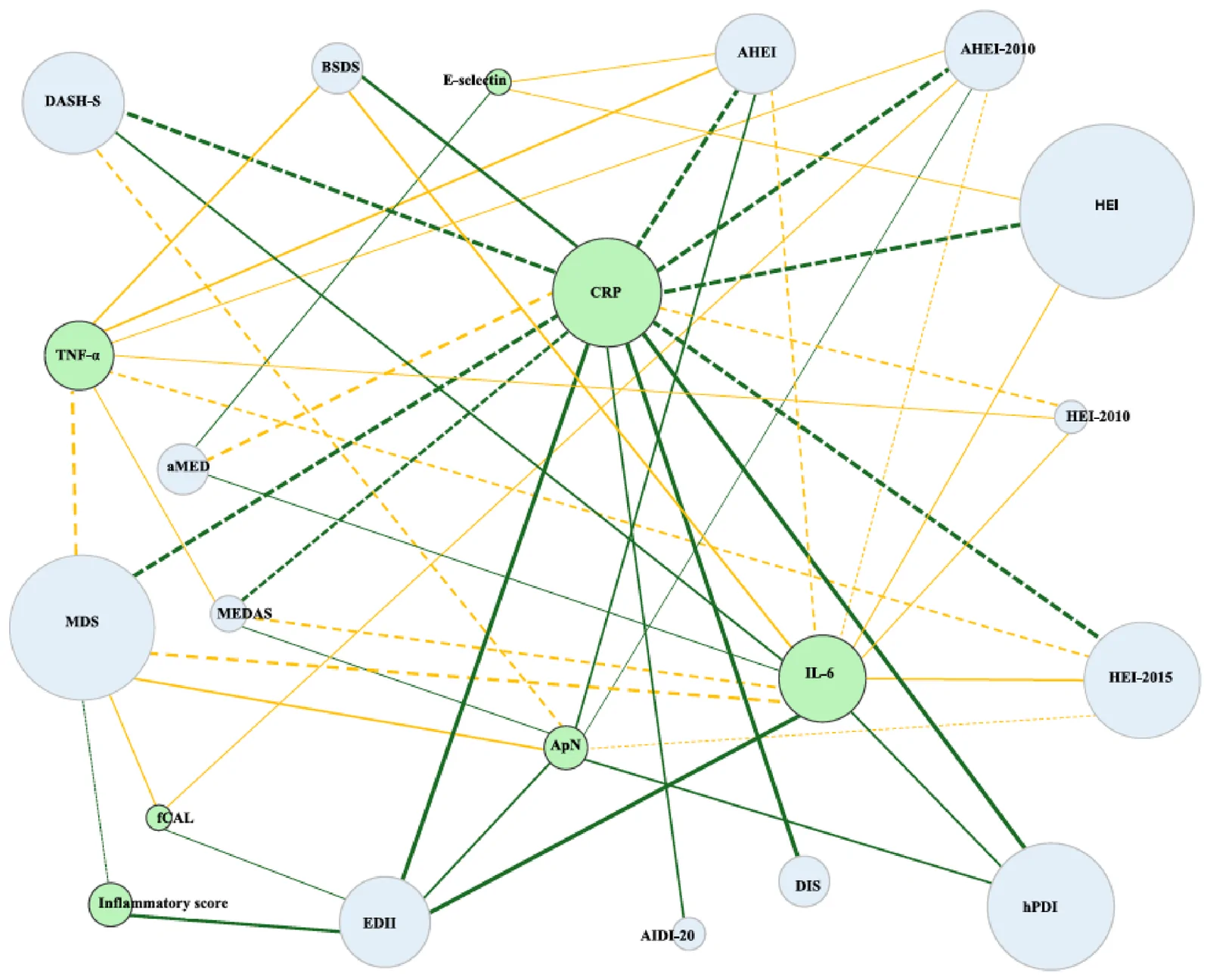
Network map depicting the reported associations between dietary indexes and inflammatory biomarkers: Blue nodes represent a dietary index, and green nodes represent an inflammatory biomarker. Node size indicates assessment extent (larger size represents more studies and larger populations). Lines denote reported associations between the index and the inflammatory marker as follows: Solid green—significant association; dotted green—mixed findings, majority showing significant association; solid yellow—no significant association; dotted yellow—mixed findings, majority showing no significant association; line thickness represents study/population size. ApN, adiponectin; CRP, high-sensitivity C-reactive protein; fCAL, fecal calprotectin; IL-6, interleukin-6; MEDAS, Mediterranean Diet Adherence Screener (from PREDIMED Study), TNFα, tumor necrosis α. Modified from [7].

Based on the above-reported considerations, it is now well established that any nutritional, nutraceutical and pharmacological strategy able to mitigate low-grade chronic inflammation, for example, resulting in inhibition of NLRP3 inflammasome activation and reduction in circulating hsCRP levels, has the potential to reduce the ASCVD burden [18]. This positive outcome should be pursued in different subsets of subjects in primary prevention and with various CVD risk, as well as in patients with high/very high CVD risk, such as patients in secondary CVD prevention and specific subgroups of patients with genetic diseases, like familial hypercholesterolemia (FH) [32], even though they are under pharmacological treatment [33]. In this direction, nutritional strategies appear very effective and powerful for several reasons, including: 1. the mandatory nature of our daily food intake, which therefore can be oriented toward anti-inflammation; 2. the multicomponent structure of diet, which should result in a balanced and effective modulation of the low-grade inflammatory process over time (and hopefully be lifelong, when applicable) and being not associated with relevant adverse effects, although tolerability and suitability may vary according to the dietary pattern and patient characteristics; and 3. the recent development and validation of original dietary inflammatory indexes [7]. These scoring tools include the Dietary Inflammatory Index (DII), the Empirical Dietary Inflammatory Pattern (EDIP), and the Anti-Inflammatory Diet Index (AIDI), and may support a more precise definition of the anti-inflammatory activity of a specific diet. These indexes grade food parameters based on their proven effects on systemic inflammatory biomarkers (extensively reviewed in [7]). This review paper by Reyneke et al. [7] aimed to synthesize the literature on food-based diet quality indexes and their association with chronic inflammation by assessing 43 food-based indexes (65 studies) categorized into four distinct groups based on dietary patterns, dietary guidelines, dietary inflammatory potential, and therapeutic diets. Established indexes based on the Mediterranean Diet (MedDiet) and dietary guidelines were mainly utilized, showing inverse associations with several inflammatory biomarkers (Figure 2). Most studies (71%) explicitly included inflammation as a key outcome, utilizing CRP or hsCRP as the most frequently evaluated inflammatory biomarker. Moreover, 57% of the studies assessed the association between dietary index and multiple inflammatory biomarkers and/or an inflammatory biomarker score (Figure 2). AIDI, Dietary Inflammation Score, and EDIP were identified as robust, empirically derived indexes to assess diet quality based on their inflammatory potential. The dietary composition of the evaluated indexes ranged from 4 to 28 dietary components, with fruits, vegetables, whole grains, and legumes consistently classified as favorable, whereas red/processed meats and added sugars were unfavorable. More work seems necessary, however, in the future to overcome the methodological variations and the inconsistencies in algorithms, promoting the use of validated inflammatory biomarkers, possibly incorporated into composite biomarker scores. Such complexity and uncertainty are depicted in Figure 2, with hsCRP being, so far, the only clinically validated biomarker for low-grade chronic inflammation and the most studied biomarker, showing, however, some substantially mixed evidence for several dietary patterns.

Interestingly, despite methodological challenges in developing and applying dietary indexes, particularly in establishing appropriate cut-off thresholds, a relevant consistency is represented by the significant intercorrelations that were observed among food-based indexes in terms of anti-inflammatory properties. This suggests that multiple indexes may reliably assess the diet–inflammation relationship, potentially offering comprehensive and generalizable considerations across different populations. On the other side, inconsistencies in the classification and categorization of some dietary components highlight a common challenge in nutrition research, as observed, for example, for legumes, different food combinations, and cooking and processing methods, which may influence the relationship between dietary patterns and inflammatory biomarkers [7]. Moreover, in addition to the NLRP3 inflammasome gene variability mentioned above, the impact of additional genetic variability in the (anti- or pro-)inflammatory response to dietary intervention should also be considered as a relevant factor of inconsistency within some studies or between different studies. Thus, as a general practical consideration, while anti-inflammatory dietary substitutions improve biomarkers irrespective of some gene variants, genotype-informed nutrition seems to yield the largest absolute risk reduction in high-risk populations. Therefore, the future clinical approach should combine, for these subjects, baseline diet-quality guidance with some validated targeted strategies for genotype-specific response patterns (e.g., APOA2 antioxidant heterogeneity or TCF7L2 carbohydrate thresholds) to improve efficacy at the individual level [28].

Overall, according to this scoping review [7], the optimal tool for assessing dietary quality in relation to chronic inflammation remains unclear, despite important efforts, and the applicability of these indexes appears more useful to validate specific nutritional strategies or nutritional guidelines, rather than to be used in the clinical practice, at least for the time being. The subsequent assessment of reduction in MACEs, which is the ultimate goal of the anti-inflammatory dietary intervention, remains, however, to be performed for most studies.

In the following section we discuss the most established nutritional patterns in relationship with mitigation of low-grade chronic inflammation and, when assessed, ASCVD risk prevention and MACE reduction. The MedDiet is a dietary pattern originated over millennia in the Mediterranean basin and complemented by behavioral habits. Over the last four decades, several relevant studies highlighted the positive impact of the MedDiet on the onset and progression of cardiovascular and metabolic diseases [34]. Such evidence can be traced back to the initial observational studies by Ancel Keys (Seven Country Study) on incidence and mortality for CVD and include, among the others, randomized trials like the Lyon Diet Heart Study (a randomized secondary prevention trial aimed at testing the preventive role of MedDiet for recurrence after a first myocardial infarction) [34], and the PREDIMED study on cardiovascular outcomes [35,36]. The findings of these trials showed that the beneficial effects of the MedDiet on cardiovascular and metabolic disorders could be mainly related to its anti-inflammatory and antioxidant properties, with specific modulation of low-grade chronic inflammation [34,37]. Table 1 reports the changes in specific circulating biomarkers, including hsCRP, observed in different dietary intervention trials with cardiovascular outcomes. Interestingly, higher adherence to a MedDiet plan was also associated with better low-grade inflammation and improved dyslipidemia in FH patients [32]. Several key compounds present in the MedDiet, like polyphenols, including molecules like oleuropein, resveratrol, flavonoids and catechins, and downregulate NF-kB signaling, thereby decreasing circulating levels of hsCRP, as well as of IL-6, TNF-α [38]. Moreover, the PREDIMED trial showed that a MedDiet plan supplemented with either nuts or extra virgin olive oil results in reduced cardiovascular events [39], also in association with reduction in hsCRP level [40]. Importantly, the PREDIMED trial was retracted and immediately republished to correct protocol deviations and statistical errors related to randomization, which included enrolling entire households without randomization or misapplying randomization tables. While the statistical re-analysis led to unchanged core conclusions with a roughly 30% relative risk reduction in major cardiovascular events in the two intervention arms, this case further highlighted the critical issues and limitations that are often associated with dietary intervention studies and are also related to lack of robustness of some tools (e.g., food frequency questionnaires, 24 h recall) used to assess compliance and adherence. Overall, while the evidence of the anti-inflammatory benefit of a good adherence to a MedDiet-based nutrition is quite robust, the real-world situation speaks differently, with a very scattered and poor adherence to MedDiet sometimes according to age, social condition, geographical situation and more [41,42].

The Dietary Approaches to Stop Hypertension (DASH) diet is characterized by high consumption of fruits, vegetables, whole grains, and low-fat dairy products, and limited intake of saturated fats, cholesterol, and refined sugars [44,45]. The DASH diet is suitable for long-term health management due to its advantages of standardized regimens and multi-target metabolic regulation. The phytochemicals (phytosterols, flavonoids, carotenoids) have been shown to promote several health benefits for cardiovascular health, including reduction in low-grade inflammation. DASH diet studies consistently show an inverse association with circulating IL-6 levels, while such association with hsCRP is present in the majority of, but not all, studies (Figure 2). Importantly, the DASH diet scoring system from several studies strongly correlated with other dietary/anti-inflammatory scores like the Healthy Eating Index (HEI), the Alternate Healthy Eating Index (AHEI) and the Mediterranean diet score (MDS), reinforcing their consistency in assessing inflammation-related outcomes [7].

Interestingly, positive nutritional modulation of gut microbiota is believed to be a main promoter of reduction in systemic low-grade chronic inflammation via reduction in meta-inflammation. It is well established that cardiometabolic comorbidities are associated with gut dysbiosis, loss of short-chain fatty acid (SCFA)-producing taxa, and disruption of the intestinal barrier, leading to endotoxemia and upregulation of pro-inflammatory pathways such as Toll-like receptor4 (TLR4)- and NLRP3-mediated signaling. As beneficial gut-derived metabolites (acetate, propionate, butyrate) decrease, detrimental metabolites increase (e.g., TMAO, as mentioned above). SCFAs, including acetate, propionate, and butyrate, may act as anti-inflammatory and epigenetic modulators through the inhibition of NF-κB, NLRP3, and histone deacetylases [51]. In this direction, MedDiet and DASH were found to be associated with favorable microbiota profiles, with increased abundance of *Faecalibacterium prausnitzii*, *Akkermansia muciniphila*, and *Bifidobacterium*, as well as reduction in pro-inflammatory metabolites such as lipopolysaccharides and TMAO [52]. From the point of view of clinical practice, however, the nutritional impact to positively modulate gut microbiota remains rather empirical and based upon adherence to one of the anti-inflammatory dietary strategies discussed in this review and the improvement of clinical symptoms, if present, and inflammatory biomarkers, which may be limited today to hsCRP. Indeed, the specific mapping of the microbiome before and after dietary intervention is not yet practical and remains in the research area until future developments that should also consider gut microbiota complexity in its entirety, thus including assessments of viromes, protozoa, and fungi, along with bacteria.

The ketogenic diet (KD) is an umbrella name including different dietary variants (including the Very Low-Calorie Ketogenic Diet (VLCKD) and the Very Low-Energy Ketogenic Therapy (VLEKT)), all aiming at increasing ketone body formation for a limited time and being generally characterized by low carbohydrate and high-quality fat intake, including those from olive oil and fatty fish, which provide essential fatty acids with antioxidant and anti-inflammatory properties [46,47]. It is an interesting approach to some cardiometabolic conditions; however, it is to be conducted under expert physician supervision. Multiple human and experimental studies support a role for ketosis in ameliorating low-grade inflammation by different mechanisms, including a reduction in NLRP3 inflammasome activation, with, as an important player, the main circulating ketone body beta-hydroxybutyrate (BHB), and with reduction in calculated ASCVD risk [46,47]. As a consequence, interventions that can induce ketosis (e.g., fasting, intermittent fasting, time-restricted feeding/eating, very low-calorie ketogenic diets) and/or increase circulating BHB by supplementation showed interest for their therapeutic potential by acting through multiple interacting mechanisms in a cell-, disease- and context-specific manner, with, in addition to reduced NLRP3 inflammasome activation [53], the possible contribution of several other associated metabolic responses, like suppression of the inflammatory promoter nuclear factor kappa-light-chain-enhancer of activated B cells (NF-kB) in macrophages, elevated lipolysis, low plasma insulin levels, stable plasma glucose, and negative energy balance able to impact innate immune responses [46,47]. It should be recalled that KD interventions are often associated with rapid weight loss and relevant metabolic changes and may be accompanied by reductions in short-chain fatty acid-producing bacteria, depletion of *Bifidobacterium*, and impaired gut barrier integrity, raising concerns regarding long-term microbiota resilience and thus requiring expert medical support.

Fiber-rich diets were shown to positively influence metabolic markers, including inflammatory markers, while also impacting the levels of various lipoproteins involved in lipid transport. Diets rich in food with abundant anti-inflammatory components, such as polyphenols, omega-3 fatty acids, fiber and essential micronutrients, target low-grade chronic inflammation, thereby modulating lipid metabolism, reducing oxidative stress and reducing ASCVD risk [50]. A controversial issue is represented by the consumption of food for pleasure like chocolate and wine, which is mainly associated with adverse health effects specifically on the cardiovascular system. On the other hand, if consumed in optimal amounts, they may have cardiovascular benefits. Accumulated evidence indicates that phenolic compounds, present in these foods, are characterized by anti-inflammatory, antioxidant, and antiplatelet properties, and their positive effects may be amplified by other compounds (esters, amines, biogenic amines, amino acids, fatty acids, mineral ingredients, and vitamins) present in chocolate and wine. Interestingly, in meta-analyses of intervention studies, appropriate consumption of chocolate and wine was positively associated with beneficial outcomes associated with the cardiovascular system, but with important caveats related to age, sex, body weight, and the presence of medical conditions including use of cardiovascular drugs. Thus, along with abundant evidence to prove the beneficial impact of consuming both products on cardiovascular health, some evidence remains controversial, suggesting a prudent approach to their use [54].

## 4. Nutraceutical Products and Mitigation of Low-Grade Chronic Inflammation: Potential Impact on Atherosclerotic Cardiovascular Disease Risk

Several food compounds (in part already mentioned above as dietary component) have been incorporated in concentrated form into food supplements, with the aim of modulating low-grade chronic inflammation. However, to date, no such products received a specific nutraceutical claim by either the FDA or EFSA regarding the treatment or mitigation of chronic low-grade inflammation. Even more importantly, the lack of evidence is also present relative to hard endpoints, like reduction in ASCVD [55]. Nevertheless, substantial clinical and experimental evidence supports the anti-inflammatory effects of several bioactive compounds incorporated into food supplements at high concentration, which consistently reduce circulating inflammatory biomarkers, including hsCRP, and modulate key inflammatory pathways. These products include omega-3 fatty acids, curcumin, olive oil polyphenols, dietary fiber, vitamin D and selected probiotics. It is important to mention that these are some of the most frequently utilized in different application contexts, such as primary cardiovascular and metabolic prevention, longevity modulation in recreational and professional athletes. While prospectively interesting, this field shows important inconsistencies, including lack of full standardization of the active compounds, considerable differences in formulation, association of active compounds in one formulation, dose, studied population, intervention duration, and the lack of robust long-term clinical trials validating the usefulness of specific anti-inflammatory compounds formulated as food supplements to ultimately mitigate ASCVD risk and onset, and therefore, there is a lack of strength of evidence. Such limitations may derive in part by reduced research investments in this area.

In this context, an active topic of mainly still-experimental research is the development of direct or indirect NLRP3 inhibitors, which has gained considerable interest as a potential treatment approach to ASCVD risk (co-)mitigation, offering enhanced mechanistic specificity and the potential to attenuate pathological inflammation, minimizing interference with other physiological and host-defense mechanisms [56]. In addition to a series of synthetic NLRP3 inhibitors (out of topic and not discussed here), a variety of natural products have been shown to modulate NLRP3 inflammasome activity across endothelial cells, vascular smooth muscle cells, and macrophages, thereby exerting protective effects against atherosclerosis, possibly representing an interesting area for novel clinical applications. These compounds generally act by suppressing NLRP3 expression, inhibiting caspase-1 and ASC activity, and reducing the release of inflammatory mediators. Here we mention some of the most studied in experimental settings, with their clinical application still to be validated [56]. Andrographolide, the principal active constituent of *Andrographis paniculata*, inhibits ox-LDL-induced NLRP3 inflammasome assembly in macrophages. Polydatin, a glycosylated derivative of resveratrol, regulates lipid metabolism, suppresses inflammatory mediator release, and enhances antioxidant enzyme activity. Quercetin, a natural flavonoid with antioxidant and anti-inflammatory properties, attenuates atherosclerosis progression in ApoE-deficient mice on a high-fat diet. It inhibits NLRP3 inflammasome activation in ox-LDL-loaded macrophages, reduces cellular steatosis and oxidative stress, and suppresses macrophage pyroptosis. Salvianolic acids A and B, major active components of *Salvia miltiorrhiza*, protect against atherosclerosis-related inflammation by inhibiting the NF-kB/NLRP3 pathway, reducing ROS release and modulating glycolysis and pyroptosis. Baicalein, the main flavonoid compound in *Scutellaria baicalensis*, alleviates inflammation by inhibiting NLRP3 inflammasome assembly and blocking the NLRP3/caspase-1 pathway. Oridonin effectively inhibits NLRP3 inflammasome activation in ApoB-deficient mice, thereby reducing inflammatory responses. Moreover, several traditional Chinese medicine formulas and patent medicines have been investigated for their effects on the NLRP3 inflammasome in atherosclerosis, with possible slowing of disease progression. Overall, food supplements with nutraceutical properties able to mitigate low-grade inflammation and, possibly, ASCVD onset and progression are still matters of research which will hopefully provide novel ways to manage this specific pathogenetic mechanism [54].

## 5. Clinical Implications

Based on the evidence discussed in this narrative review, a practical approach to low-grade inflammation in the clinical practice aimed at ASCVD prevention and management may be proposed. A first level approach that can be used in subjects at apparently low ASCVD risk may include 1. measurement of circulating hsCRP, 2. implementation of appropriate lifestyle including an anti-inflammatory diet and monitoring of adherence to it, and 3. reassessment of hsCRP values after an appropriate period (i.e., 3 to 6 months), all in the context of a cardiometabolic evaluation. For patients at high-/very high-ASCVD risk, an additional strategy should include a more personalized evaluation, with consideration of specific sex- and age-related issues, comorbidities, and current pharmacological therapy. In this context, the additional use of approved anti-inflammatory drugs, like low-dose colchicine, and, possibly in the future, of validated nutraceutical supplements may also be considered while waiting for more personalized approaches based on multiomics analyses and machine learning algorithms for precision medicine.

## 6. Conclusions and Future Perspectives

Given the relevance of low-grade chronic inflammation to promote ASCVD risk, in the future, it appears important to highlight the following aspects: 1. It is crucial to increase the awareness of this pathogenetic process among both healthcare providers and patients/citizens. 2. From a diagnostic angle, it is crucial to identify and validate novel biomarkers for ASCVD-related low-grade inflammation to be possibly combined into composite biomarker scores able to contribute to more accurately describing the individual ASCVD risk. 3. At least for high-risk patients, these scores may be implemented into machine learning algorithms to implement a more effective and precise strategy of precision medicine. 4. It is crucial to establish the actionability, by nutritional strategies or also by synthetic or natural compounds, of novel biomarkers leading to reduction in their levels, and more importantly, to recognize that actual mitigation of ASCVD risk and occurrence is of utmost relevance. 5. As the last segment linked to this path, future studies will need to implement novel effective approaches and components related to nutrition strategies, as well as the use of selected nutraceutical products and food supplements [57]. While dietary approaches have been validated at least in part regarding their anti-inflammatory activity and, in a few cases, also for their positive effect on ASCVD risk, food supplements/nutraceuticals, although interesting for specific applications, still await full experimental and especially clinical validation [55]. 6. A challenging methodological context is well known to be intrinsic to nutritional research, since it shows peculiar issues that include, among many, the lack of a true placebo arm in randomized trials, the critical aspect of adherence, the cultural and ethnic habits and their changes over individual lifetime, and the resistance to change/improve a consolidated nutritional profile.

## Figures and Tables

**Table 1:** Nutritional interventions effects (↑ increase; ↓ decrease) on low-grade chronic inflammation and other biomarkers of atherosclerotic cardiovascular disease.

Dietary Pattern	Low-Grade Inflammation	Lipid Biomarkers	Other Cardiometabolic Markers	Refs.
**Mediterranean Diet**	↓ hsCRP, ↓ IL-6, ↓ TNF-α	↓ TC, ↓ LDL-C, ↓ ApoB, ↑ HDL-C, ↓ LDL oxidation	↓ ROS	[32,34,37]
**PREDIMED Diet**	↓ hs-CRP, ↓ IL-1β, ↓ IL-6, ↓ IL-8, ↓ IL-12p70, ↓ TNF-α, ↓ MCP-1	↑ HDL-C, ↓ LDL-C, ↓ TG	↓ Hypertension, ↑ insulin sensitivity	[35,43]
**DASH Diet**	↓ Inflammation (not all studies)	↓ LDL-C, ↓ TC,	↓ Hypertension, ↓endothelial dysfunction	[44,45]
**Ketogenic Diet**	↓ hs-CRP	↓ TG, ↑ HDL-C	Improved insulin sensitivity, some risk of elevated LDL-C	[46,47]
**Plant-Based Diet**	↓ Inflammation	↓ TC, ↓ LDL-C, ↓ ApoB, ↑ ApoA1	Improved ApoB/ApoA1 ratio, ↑ antioxidant activity	[48,49]
**High-Fiber Diet**	↓ Systemic inflammation	↓ TC, ↓ LDL-C	↑ Insulin sensitivity	[50]
**Anti-inflammatory Diet**	↓ Chronic inflammation	Modulation of lipid metabolism	↓ Oxidative stress, enhanced lipid regulation	[50]

## Data Availability

No new data were created or analyzed in this study. Data sharing is not applicable to this article.

## References

[1] Stark B.A., Decleene N.K., Desai E.C., Hsu J.M., Johnson C.O., Lara-Castor L., Legrand K.E., Bhoomadevi A., Aalipour M.A., Aalruz H. (2025). Global, Regional, and National Burden of Cardiovascular Diseases and Risk Factors in 204 Countries and Territories, 1990–2023. JACC.

[2] Lloyd-Jones D.M., Allen N.B., Anderson C.A.M., Black T., Brewer L.C., Foraker R.E., Grandner M.A., Lavretsky H., Perak A.M., Sharma G. (2022). Life’s Essential 8: Updating and Enhancing the American Heart Association’s Construct of Cardiovascular Health: A Presidential Advisory from the American Heart Association. Circulation.

[3] Munjas J., Vladimirov S., Ratkovic T., Comi L., Giglione C., Tanaskovic I., Gojkovic T., Rakic B., Davidovic A., Vukmirovic L. (2025). Toward Precision Medicine in Atherosclerotic Cardiovascular Disease: Insights from Omics Data into Sex Differences. Curr. Atheroscler. Rep..

[4] Gerdts E., Novella S., Devaux Y., Magni P., Marti H.P., Sopić M., Kararigas G. (2025). Biological Sex and Cardiovascular Disease Prevention in Systemic Arterial Hypertension. Arterioscler. Thromb. Vasc. Biol..

[5] Mombelli G., Bosisio R., Calabresi L., Magni P., Pavanello C., Pazzucconi F., Sirtori C.R. (2015). Gender-related lipid and/or lipoprotein responses to statins in subjects in primary and secondary prevention. J. Clin. Lipidol..

[6] Adamstein N.H., Moorthy M.V., Cook N.R., Buring J.E., Sesso H.D., Ridker P.M. (2026). Inflammation and the Long-Term Incidence of Hypertension in Women. Eur. J. Prev. Cardiol..

[7] Reyneke G.L., Lambert K., Beck E.J. (2025). Food-based indexes and their association with dietary inflammation. Adv. Nutr..

[8] Nanri H., Nakamura K., Hara M., Higaki Y., Imaizumi T., Taguchi N., Sakamoto T., Horita M., Shinchi K., Tanaka K. (2011). Association Between Dietary Pattern and Serum C-Reactive Protein in Japanese Men and Women. J. Epidemiol..

[9] Hageman S., Pennells L., Ojeda F., Kaptoge S., Kuulasmaa K., de Vries T., Xu Z., Kee F., Chung R., Wood A. (2021). SCORE2 risk prediction algorithms: New models to estimate 10-year risk of cardiovascular disease in Europe. Eur. Heart J..

[10] Usova E.I., Alieva A.S., Yakovlev A.N., Alieva M.S., Prokhorikhin A.A., Konradi A.O., Shlyakhto E.V., Magni P., Catapano A.L., Baragetti A. (2021). Integrative Analysis of Multi-Omics and Genetic Approaches—A New Level in Atherosclerotic Cardiovascular Risk Prediction. Biomolecules.

[11] Nordestgaard L.T., Wolford B.N., de Gonzalo-Calvo D., Sopić M., Devaux Y., Matic L., Wettinger S.B., Schmid J.A., Amigó N., Masana L. (2025). Multiomics in atherosclerotic cardiovascular disease. Atherosclerosis.

[12] Sopic M., Vilne B., Gerdts E., Trindade F., Uchida S., Khatib S., Wettinger S.B., Devaux Y., Magni P. (2023). EU-AtheroNET COST Action CA21153. Multiomics tools for improved atherosclerotic cardiovascular disease management. Trends Mol. Med..

[13] Nordestgaard L.T., Magni P., Sopić M., Chemaly M., Matic L., Dalila N., Trindade F., Wolford B.N., Rodriguez-Hernandez Z., Amigó N. (2026). Multiomics for Risk Stratification in Atherosclerotic Cardiovascular Disease. Circ. Genom. Precis. Med..

[14] Ross R., Neeland I.J., Yamashita S., Shai I., Seidell J., Magni P., Santos R.D., Arsenault B., Cuevas A., Hu F.B. (2020). Waist circumference as a vital sign in clinical practice: A Consensus Statement from the IAS and ICCR Working Group on Visceral Obesity. Nat. Rev. Endocrinol..

[15] Neeland I.J., Ross R., Després J.P., Matsuzawa Y., Yamashita S., Shai I., Seidell J., Magni P., Santos R.D., Arsenault B. (2019). Visceral and ectopic fat, atherosclerosis, and cardiometabolic disease: A position statement. Lancet Diabetes Endocrinol..

[16] Mach F., Koskinas K.C., Roeters van Lennep J.E., Tokgözoğlu L., Badimon L., Baigent C., Benn M., Binder C.J., Catapano A.L., De Backer G.G. (2025). 2025 Focused Update of the 2019 ESC/EAS Guidelines for the management of dyslipidaemias. Atherosclerosis.

[17] Biolo M., Portinari C., Goffi C., Berti R., Bertocco S., Previato L., Zambon S., Simioni P., Zambon A. (2026). Focus on lipoprotein(a). Eur. Heart J. Suppl..

[18] Mensah G.A., Arnold N., Prabhu S.D., Ridker P.M., Welty F.K. (2026). Inflammation and Cardiovascular Disease: 2025 ACC Scientific Statement. JACC.

[19] Markus M.R.P., Ittermann T., Mariño Coronado J., Schipf S., Bahls M., Könemann S., Chamling B., Völzke H., Damasceno N.R.T., Santos R.D. (2025). Low-density lipoprotein cholesterol, lipoprotein(a) and high-sensitivity C-reactive protein are independent predictors of cardiovascular events. Eur. Heart J..

[20] Magni P., Bier D.M., Pecorelli S., Agostoni C., Astrup A., Brighenti F., Cook R., Folco E., Fontana L., Gibson R.A. (2017). Perspective: Improving Nutritional Guidelines for Sustainable Health Policies: Current Status and Perspectives. Adv. Nutr..

[21] Martinon F., Burns K., Tschopp J. (2002). The Inflammasome. Mol. Cell.

[22] Curia M.C., Pignatelli P., D’Antonio D.L., D’Ardes D., Olmastroni E., Scorpiglione L., Cipollone F., Catapano A.L., Piattelli A., Bucci M. (2022). Oral *Porphyromonas gingivalis* and *Fusobacterium nucleatum* Abundance in Subjects in Primary and Secondary Cardiovascular Prevention, with or without Heterozygous Familial Hypercholesterolemia. Biomedicines.

[23] Ricciardi R.M., Cipollone A., D’Ardes D., Di Giacomo D., Pignatelli P., Cipollone F., Curia M.C., Magni P., Bucci M. (2023). Risk Factors and Immunoinflammatory Mechanisms Leading to Atherosclerosis: Focus on the Role of Oral Microbiota Dysbiosis. Microorganisms.

[24] Baragetti A., Catapano A.L., Magni P. (2020). Multifactorial Activation of NLRP3 Inflammasome: Relevance for a Precision Approach to Atherosclerotic Cardiovascular Risk and Disease. Int. J. Mol. Sci..

[25] Wang Z., Hu W., Lu C., Ma Z., Jiang S., Gu C., Acuña-Castroviejo D., Yang Y. (2018). Targeting NLRP3 (Nucleotide-Binding Domain, Leucine-Rich–Containing Family, Pyrin Domain–Containing-3) Inflammasome in Cardiovascular Disorders. Arterioscler. Thromb. Vasc. Biol..

[26] Zhang T., Luo L., Yang K., Liu H., Xiao S., Wang M., Chen J., Zhou Y., Ge L. (2026). NLRP3 inflammasome in cardiovascular diseases: Molecular mechanisms and advances in targeted drug research. Front. Immunol..

[27] Scalabrino G., Corsi M.M., Veber D., Buccellato F.R., Pravettoni G., Manfridi A., Magni P. (2002). Cobalamin (vitamin B12) positively regulates interleukin-6 levels in rat cerebrospinal fluid. J. Neuroimmunol..

[28] Liu X., Xu M., Wang H., Zhu L. (2025). Integrating Precision Medicine and Digital Health in Personalized Weight Management: The Central Role of Nutrition. Nutrients.

[29] Mehta A., Blumenthal R.S., Gluckman T.J., Feldman D.I., Kohli P. (2025). High-sensitivity C-reactive Protein in Atherosclerotic Cardiovascular Disease: To Measure or Not to Measure?. US Cardiol. Rev..

[30] Nordestgaard A.T., Ridker P.M., Nordestgaard B.G. (2026). Cardiovascular risk in inflamed Danish women and men without standard modifiable risk factors. Eur. J. Prev. Cardiol..

[31] Ridker P.M. (2016). A Test in Context: High-Sensitivity C-Reactive Protein. J. Am. Coll. Cardiol..

[32] Antoniazzi L., Arroyo-Olivares R., Bittencourt M.S., Tada M.T., Lima I., Jannes C.E., Krieger J.E., Pereira A.C., Quintana-Navarro G., Muñiz-Grijalvo O. (2021). Adherence to a Mediterranean diet, dyslipidemia and inflammation in familial hypercholesterolemia. Nutr. Metab. Cardiovasc. Dis..

[33] Averna M., Banach M., Bruckert E., Drexel H., Farnier M., Gaita D., Magni P., März W., Masana L., Mello E Silva A. (2021). Practical guidance for combination lipid-modifying therapy in high- and very-high-risk patients: A statement from a European Atherosclerosis Society Task Force. Atherosclerosis.

[34] Finicelli M., Di Salle A., Galderisi U., Peluso G. (2022). The Mediterranean Diet: An Update of the Clinical Trials. Nutrients.

[35] Medina-Remón A., Casas R., Tressserra-Rimbau A., Ros E., Martínez-González M.A., Fitó M., Corella D., Salas-Salvadó J., Lamuela-Raventos R.M., Estruch R. (2017). Polyphenol intake from a Mediterranean diet decreases inflammatory biomarkers related to atherosclerosis: A substudy of the PREDIMED trial. Br. J. Clin. Pharmacol..

[36] Ros E., Martínez-González M.A., Estruch R., Salas-Salvadó J., Fitó M., Martínez J.A., Corella D. (2014). Mediterranean Diet and Cardiovascular Health: Teachings of the PREDIMED Study. Adv. Nutr..

[37] Richardson L.A., Izuora K., Basu A. (2022). Mediterranean Diet and Its Association with Cardiovascular Disease Risk Factors: A Scoping Review. Int. J. Environ. Res. Public Health.

[38] Upadhyay S., Dixit M. (2015). Role of Polyphenols and Other Phytochemicals on Molecular Signaling. Oxid. Med. Cell. Longev..

[39] Estruch R., Ros E., Salas-Salvadó J., Covas M.I., Corella D., Arós F., Gómez-Gracia E., Ruiz-Gutiérrez V., Fiol M., Lapetra J. (2018). Primary Prevention of Cardiovascular Disease with a Mediterranean Diet Supplemented with Extra-Virgin Olive Oil or Nuts. N. Engl. J. Med..

[40] Urpi-Sarda M., Casas R., Sacanella E., Corella D., Andrés-Lacueva C., Llorach R., Garrabou G., Cardellach F., Sala-Vila A., Ros E. (2021). The 3-Year Effect of the Mediterranean Diet Intervention on Inflammatory Biomarkers Related to Cardiovascular Disease. Biomedicines.

[41] Mattavelli E., Olmastroni E., Bonofiglio D., Catapano A.L., Baragetti A., Magni P. (2022). Adherence to the Mediterranean Diet: Impact of Geographical Location of the Observations. Nutrients.

[42] Mattavelli E., Olmastroni E., Casula M., Grigore L., Pellegatta F., Baragetti A., Magni P., Catapano A.L. (2023). Adherence to Mediterranean Diet: A Population-Based Longitudinal Cohort Study. Nutrients.

[43] Martínez-González M.A., Salas-Salvadó J., Estruch R., Corella D., Fitó M., Ros E. (2015). Benefits of the Mediterranean Diet: Insights from the PREDIMED Study. Prog. Cardiovasc. Dis..

[44] Chiavaroli L., Viguiliouk E., Nishi S.K., Blanco Mejia S., Rahelić D., Kahleová H., Salas-Salvadó J., Kendall C.W., Sievenpiper J.L. (2019). DASH Dietary Pattern and Cardiometabolic Outcomes: An Umbrella Review of Systematic Reviews and Meta-Analyses. Nutrients.

[45] Liu K., Liu S., Wang D., Qiao H. (2025). The DASH diet in diabetes related complications or comorbidities: An unexpected friend. Front. Nutr..

[46] Gershuni V.M., Yan S.L., Medici V. (2018). Nutritional Ketosis for Weight Management and Reversal of Metabolic Syndrome. Curr. Nutr. Rep..

[47] Tragni E., Vigna L., Ruscica M., Macchi C., Casula M., Santelia A., Catapano A.L., Magni P. (2021). Reduction of Cardio-Metabolic Risk and Body Weight through a Multiphasic Very-Low Calorie Ketogenic Diet Program in Women with Overweight/Obesity: A Study in a Real-World Setting. Nutrients.

[48] Feng S., Jiang Y., Jiang J., Bian H., Zhu R. (2026). Effects of diet-modulated gut microbiota and microbial metabolites in atherosclerosis. Biomed. Pharmacother..

[49] Kuchta A., Lebiedzińska A., Fijałkowski M., Gałąska R., Kreft E., Totoń M., Czaja K., Kozłowska A., Ćwiklińska A., Kortas-Stempak B. (2016). Impact of plant-based diet on lipid risk factors for atherosclerosis. Cardiol. J..

[50] Yu X., Pu H., Voss M. (2024). Overview of anti-inflammatory diets and their promising effects on non-communicable diseases. Br. J. Nutr..

[51] Li Y., Zhu J., Huang M., Liu X., Wang L. (2026). Microbiota–innate immune crosstalk drives atherosclerosis: Mechanisms, disease progression, and emerging therapeutic strategies. Front. Immunol..

[52] Onu A., Tutu A., Trofin D.M., Onu I., Galaction A.I., Onita C.A., Iordan D.A., Matei D.V. (2026). Diet, Physical Exercise, and Gut Microbiota Modulation in Metabolic Syndrome: A Narrative Review. Life.

[53] Neudorf H., Little J.P. (2024). Impact of fasting & ketogenic interventions on the NLRP3 inflammasome: A narrative review. Biomed. J..

[54] Sperkowska B., Murawska J., Przybylska A., Gackowski M., Kruszewski S., Durmowicz M., Rutkowska D. (2021). Cardiovascular Effects of Chocolate and Wine—Narrative Review. Nutrients.

[55] U.S. Food and Drug Administration Questions and Answers on Dietary Supplements. FDA. https://www.fda.gov/food/information-consumers-using-dietary-supplements/questions-and-answers-dietary-supplements.

[56] Zhen D., Liu C., Sun X., Bao T., Wang B., Zheng K., Wu Z., Chen X., Zhang G. (2026). Direct NLRP3 inflammasome inhibitors for cardiovascular therapeutics: A comparative review of synthetic and natural candidates. Front. Pharmacol..

[57] Magni P. (2023). The sex-associated burden of atherosclerotic cardiovascular diseases: An update on prevention strategies. Mech. Ageing Dev..

